# Adaptation and validation of the Caregiver Burden Inventory for use with caregivers of elderly individuals^1^

**DOI:** 10.1590/0104-1169.3357.2534

**Published:** 2015

**Authors:** Daiany Borghetti Valer, Marinês Aires, Fernanda Lais Fengler, Lisiane Manganelli Girardi Paskulin

**Affiliations:** 2 MSc; 3 Doctoral student, Universidade Federal do Rio Grande do Sul, Porto Alegre, RS, Brazil. Professor, Universidade Regional Integrada do Alto Uruguai e das Missões, Porto Alegre, RS, Brazil; 4 Undergraduate student in Nursing, Escola de Enfermagem, Universidade Federal do Rio Grande do Sul, Porto Alegre, RS, Brazil; 5 PhD, Adjunct Professor, Escola de Enfermagem, Universidade Federal do Rio Grande do Sul, Porto Alegre, RS, Brazil

**Keywords:** Caregivers, Aged, Nursing, Validation Studies

## Abstract

**OBJECTIVE:**

to adapt and validate the Caregiver Burden Inventory for use with caregivers of older adults in Brazil.

**METHOD:**

methodological study involving initial translation, synthesis of translations, back translation, expert committee review, pre-testing, submission of the final version to the original authors, and assessment of the inventory's psychometric properties. The inventory assesses five dimensions of caregiver burden: time-dependence, developmental, physical, social and emotional dimensions.

**RESULTS:**

a total of 120 family caregivers took part in the study. All care-receivers were older adults dependent on assistance to perform activities of daily living, and lived in the central region of the city of Porto Alegre, RS, Brasil. Cronbach's alpha value for the inventory was 0.936, and the Pearson correlation coefficient for the relationship between the scores obtained on the Caregiver Burden Inventory and the Burden Interview was 0.814. The intraclass correlation coefficient was 0.941, and the value of Student's T-test comparing test and retest scores was 0.792.

**CONCLUSION:**

the instrument presented adequate reliability and the suitability of its items and factors was confirmed in this study.

## Background

The process of transcultural adaptation involves the development of versions of an assessment instrument that are equivalent to the original, but at the same time linguistically and culturally adapted to a different context than the original. Therefore, the adaptation of assessment instruments allows comparisons among results of investigations conducted in different cultures, aiding the exchange of information within the international scientific community, and decreasing costs and time spent in the process^(^ 1 ^)^. 

The Caregiver Burden Inventory was developed by Canadian researchers and has already been adapted to Chinese^(^ 2 ^)^. The authors of the instrument stress the importance of using a multidimensional measurement of burden with separate scores for each dimension in as opposed to global or uni-dimensional scores. Global scores tend to mask differences between the levels of burden on different domains of caregiving, and do not allow for the investigation of caregiver burden profiles^(^ 3 ^)^. There is no consensus concerning the conceptualization of "caregiver burden" in the literature and the expression "caregiver burden" in this study refers to the physical, psychological, emotional, social and financial problems experienced by family caregivers^(^ 4 ^)^. 

 Three related assessment instruments have been adapted to the Brazilian population: the Caregiver Burden Scale^(^ 5 ^)^, the Burden Interview^(^ 6 ^)^ and the Family Burden Interview Schedule^(^ 7 ^)^. The Caregiver Burden Inventory is distinct from these three instruments in terms of the following: target population; dimensions assessed; the scoring method (global score or multidimensional scores). The Burden Interview^(^ 6 ^)^ is the most frequently used instrument in the assessment of caregiver burden in Brazilian research, and is somewhat similar to the instrument adapted by this study. Nonetheless, unlike the other two instruments adapted to the Brazilian population and the Caregiver Burden Inventory, the Burden Interview only provides a uni-dimensional, global assessment of caregiver burden. Furthermore, the Burden Interview was developed and validated exclusively for use among caregivers of older individuals with dementia. Therefore, an adaptation of the Caregiver Burden Inventory will allow the assessment of a broader caregiver sample, and the identification of the domains in which there is greater caregiver burden. 

Population aging may lead to a greater number of individuals suffering with physical and emotional disorders, increasing the demand for caregivers. A person is considered a caregiver if s/he provides in-home care for the elderly person, regardless of remuneration or degree of relationship to the care-recipient^(^ 8 ^)^. Caregivers can be either formal or informal. Informal caregivers provide unpaid care and have no professional training, which differs from formal caregivers. Examples of informal caregivers are family members, friends or neighbors^(^ 9 ^)^.

Nurses play a key role in assisting older adult caregivers, as they assess the vulnerability of caregiving situations and conduct interventions to minimize the negative influence of caregiving on well-being, effectively attending to and preserving caregiver health. 

The adaptation of the Caregiver Burden Inventory will result in an instrument that is easy to administer and score, is short and easily comprehensible and assesses a wide range of possible caregiving problems^(^ 2 ^)^. It is, therefore, a comprehensive instrument that will be useful both to health care workers and to the scientific community, allowing international score comparisons to be made among countries where this scale is already available.

The goal of this study was to adapt and validate the Caregiver Burden Inventory to be used with primary family caregivers of older adults in Brazil.

## Method

 This methodological study was conducted in the Primary Health Care Unit of the Porto Alegre *Hospital de Clínicas*, located in the central district of the city, in the state of Rio Grande do Sul, Brazil. This district has the highest percentage of older adults in the city, with individuals aged 60 years old or older making up 23.61% of its population in 2010^(^ 10 ^)^. 

The Caregiver Burden Inventory comprises 24 closed questions divided into five dimensions: time-dependence, developmental, physical, social and emotional burden. There are five items in each dimension except for physical burden, which has four items dedicated to. Each item is given a score between 0 (not at all descriptive) and 4 (very descriptive), where higher scores indicate greater caregiver burden; there are no cut-off points for classifying burden. Therefore, total scores for factors one, two, four and five can range from zero to 20. An equivalent score for physical burden can be obtained by multiplying the sum of items in this dimension by 1.25^(^ 3 ^)^.

The transcultural adaptation was carried out through the following steps: initial translation, synthesis of translations, back translation, expert committee review, pre-testing, submission of the final version to the original authors and assessment of its psychometric properties^(^ 1 ^)^.

The instrument was translated into Brazilian Portuguese by two individuals, a language teacher and a nurse, both of whom were experienced with the English language. The synthesis of these translations was produced by the translators and one of the researchers. The resulting version of the scale was then independently back-translated into English by two native English speakers with no background in the health sciences. The expert committee was composed of five health care workers and/or faculty members with experience in primary health care, as well as older adults health and home care. Each professional signed an informed consent form. The participants were fluent in English and two of these individuals have lived in English-speaking countries and three had experience with the adaptation of assessment instruments. The committee assessed the semantic, idiomatic, experiential and conceptual equivalence between the two versions and developed a pre-final version in Portuguese based on group consensus. The minimum inter-expert agreement level for the two versions was 80%. This version was submitted to pre-testing with a sample of eight caregivers. The final version was back-translated, then sent to and subsequently approved by the original author of the Canadian instrument.

An assessment of the psychometric properties, reliability and validity of the instrument was conducted to verify the applicability of the present findings to the target population. The expert committee agreed that the instrument could adequately and comprehensively measure the construct it was intended to assess, providing evidence for content validity. Concurrent validity was assessed through the Pearson correlation coefficient between the present scale and the Burden Interview. Internal consistency, determined by intercorrelations among items in the instrument, was analyzed using Cronbach's alpha. Test-retest analyses were conducted with paired t tests; Intraclass Correlation Coefficients (ICC) were used to assess the stability of scores over time^(^ 11 ^)^. 

Sample size was set at five subjects per item of the adapted instrument^(^ 12 ^)^ , so that the final sample comprised 120 primary family caregivers. These caregivers were invited to participate in the study. These caregivers were individuals who self-reported as the primary caregiver for an elderly relative over the age of 60 where the relative was identified as dependent on the caregiver to provide assistance for at least one or more activities of daily living. Exclusion criteria were caregivers under 18 years of age or who could not be contacted by phone after three attempts on different days and at different time.

To assess test-retest reproducibility, the scale was re-administered to all odd-numbered participants (n=60) 14 days after the initial application. Differences of at least one point in scores ranging between zero and 20, with a standard deviation of six points, were considered significant at 5% with statistical power of 80%. Data was collected in 2012, either at the homes of participants or the primary health care unit. Participants completed a socio-demographic information questionnaire, the Burden Interview for criterion-related validity, and the Physical Activities of Daily Living (PADL) and Instrumental Activities of Daily Living (IADL) scales to assess the elderly care receivers' functional capacity. These scales have been used in many Brazilian studies, but only limited psychometric data are available for the Brazilian translations. In 1987, the first Brazilian study of both scales was conducted; the overall Cronbach's alpha coefficient for the instrument was 0.880^(^ 13 ^)^. The Burden Interview is composed of 22 questions, with a total score ranging between zero and 88 points, where larger scores indicate more significant burden^(^ 6 ^)^. The activities of daily living scales were completed by the caregivers to assess the elderly individuals' levels of dependence. Both the PADL and IADL have a maximum score of 14 points, where higher scores indicate greater independence. 

Statistical analyses were conducted using the Statistical Package for the Social Sciences, version 17.0. Continuous variables were expressed by mean ± standard deviation, or median and interquartile ranges. Categorical variables were expressed by absolute or relative frequencies. Internal consistency was calculated using Cronbach's alpha. Student`s t test and the ICC were used to assess the test-retest reproducibility of the instrument. Correlations between scores in the Caregiver Burden Inventory and the Burden Interview were analyzed through the Pearson correlation coefficient. A Confirmatory Factor Analysis was performed using the following fitness measures: the Root Mean Square Error of Approximation (RMSEA); the Comparative Fit Index (CFI); and the Parsimony Comparative Index (PGFI). The following were considered to be cut off points: ≤0.10 for RMSEA, ≥0.90 for CFI and ≥0.60 for PGFI^(^ 14 ^)^. Lastly, the Pearson correlation coefficients were also used to investigate the relationship between the PADL and the IADL and the Caregiver Burden Inventory. Scores per domain and total score were used for analysis.

This study was approved by the Research Ethics Committee of the Porto Alegre *Hospital de Clínicas* (protocol No. 110024), and the author of the original instrument authorized its adaptation to Brazilian Portuguese. 

## Results

Participants in the study were 58.63 ±13.73 years old on average, and most individuals were female (73.3%). The mean number of years of education among participants was 12.20 ±5.44, and a total of 48.3% of participants were married. In regard to their occupation, 37.5% were retired, and 16.66% were homemakers. A total of 5% of caregivers were also formally employed. Most primary caregivers were the children of the older adults they cared for (60.8%), while 20% were the spouses of the care-recipients. Additionally, 75.8% of caregivers lived with the older person they cared for. 

The median time participants spent as caregivers was 7.56 years. Outside help was available for most participants (78%), mainly from hired caregivers. Notably, 35% of participants were 24-hour caregivers. As for financial help, 90.8% of the care-receivers had their own source of income, with 45% receiving one times the minimum wage. Lastly, 60% of the caregivers reported spending their own funds to cover caregiving costs. 

**Figure 1 -:**
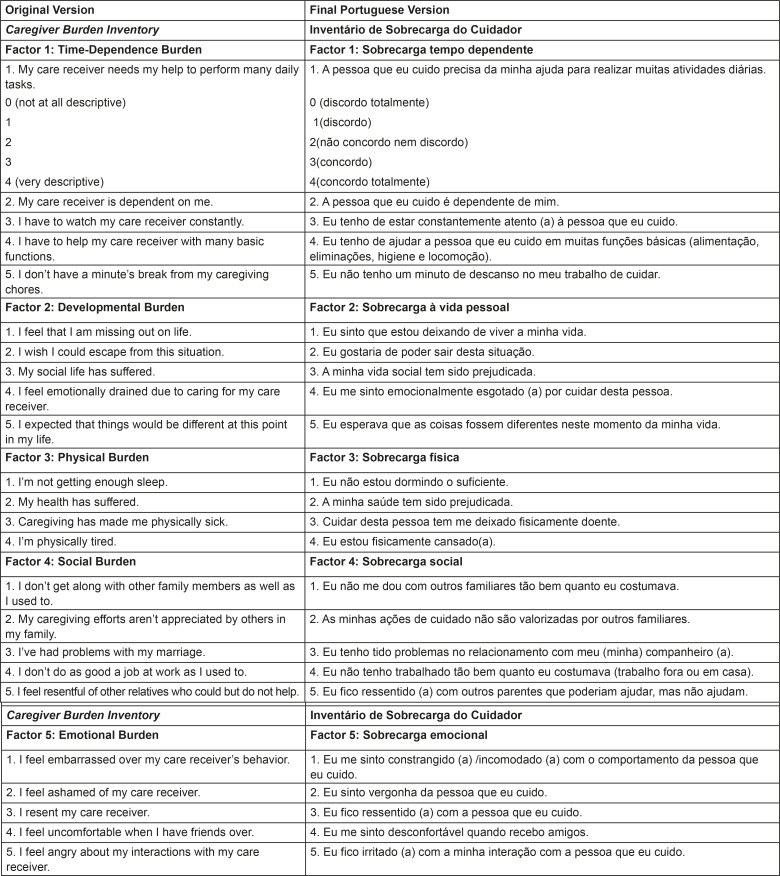
Original and adapted versions of the Caregiver Burden Inventory

In relation to the transcultural adaptation and validation, the final Portuguese version of this instrument as developed by the expert committee is presented in Figure 1.

Some modifications were made during the transcultural adaptation process. To make the questionnaire more understandable, especially with those who have had fewer years of schooling, the response descriptions were changed to "agree" and "disagree", as shown in figure 1. To increase comprehensibility of item 4 in factor 1, examples were added to the end of the sentence ("eating, toilet use, personal hygiene and locomotion") to clarify the expression "basic functions," which was translated as "*funções básicas*". Similarly, in factor 4, item 4, "(work outside or at home)" was added to the end of the sentence so it would also apply to housewives. In the original version, factor 2 is called "developmental burden." As the items in this dimension mostly refer to the personal life of the caregiver, the factor name was changed to a more colloquial expression, "personal life burden." In factor 4, item 3, in spite of the possibility of literal translation of the word "marriage" to "*casamento*", the text was modified to "relationship with my partner," so as to include people who are not legally married. Also, since there are no words for "care receiver" and "caregiving" in Brazilian Portuguese, these expressions were replaced with "the person I care for" and "caring for this person," respectively, which are more colloquial than the original items, but equivalent in meaning.

Table 1 displays Cronbach's alpha values for each factor, as well as for the total score. Cronbach's alpha values ranged between 0.753 and 0.919, with factors 2 and 3 presenting the highest values. 

**Table 1 -:** Intervals, means and Cronbach's alpha values for factors of the Caregiver Burden Inventory and total score for the sample studied (n=120). Porto Alegre, RS, Brazil, 2012.

Factor	Mean (SD)*	Cronbach’s Alpha
Factor 1 (time-dependence)	12.3 (5.0)	0.859
Factor 2 (developmental)	10.0 (6.0)	0.919
Factor 3 ( physical)	7.8(6.1)	0.911
Factor 4 ( social)	6.7 (4.9)	0.753
Factor 5 (emotional)	4.6 (4.3)	0.850
Total	41.8 (20.9)	0.936

* Standard deviation

The reproducibility of the instrument was verified for 60 caregivers. T test results (Table 2) showed no significant differences in scores between test and retest.

**Table 2 -:** Results of paired t test per inventory factor and retest, mean, standard deviation and significance and intraclass correlation coefficient. Porto Alegre, RS, Brazil, 2012

Pairs	Test score mean (Retest)	Test score SD (Retest)	p*	ICC^†^
Factor 1 - Factor 1 Retest	12.6 (12.4)	5.1 (4.9)	0.465	0.916
Factor 2 - Factor 2 Retest	9.7 (10.1)	5.9 (5.5)	0.410	0.895
Factor 3 - Factor 3 Retest	7.7 (7.4)	5.7 (5.0)	0.582	0.883
Factor 4 - Factor 4 Retest	6.8 (6.6)	4.9 (4.8)	0.731	0.867
Factor 5 - Factor 5 Retest	4.4 (4.7)	4.4 (4.3)	0.436	0.926
Test total - Retest total	41.6 (41.9)	20.8 (19.0)	0.792	0.941

* p: significance

† Intraclass Correlation Coefficient

Table 3 shows that correlation coefficients between factors in the two instruments ranged between 0 and 1, with the largest correlations observed between factors 2 and 3.

**Table 3 -:** Pearson correlation coefficients and significance of correlations between factor scores and total scores in the Caregiver Burden Inventory and the Burden Interview. Porto Alegre, RS, Brazil, 2012.

Factors CBI* – *BI* ^†^	Pearson correlation coefficient	p ^‡^
Factor 1	0.4	< 0.001
Factor 2	0.7	< 0.001
Factor 3	0.7	< 0.001
Factor 4	0.6	< 0.001
Factor 5	0.6	< 0.001
Total	0.8	< 0.001

* Caregiver Burden Inventory

† Burden Interview

‡ p: significance

Through confirmatory factor analysis, the following adjustment measures have been identified: RMSEA = 0.065, CFI = 0.935 and PGFI = 0.645. 

The mean score on the Caregiver Burden Inventory was 41.80 ±20.99. The highest scores were observed in the time-dependence burden factor (12.36 points). Mean scores on the Burden Interview were 29.53 ±15.13. The mean functional capacity scores for the care-receivers were 6.3 for the IADL and 10.53 for the PADL. The correlation coefficient of -0.5 between total caregiver burden scores and the care-recipients' functional capacity indicated that higher levels of care-recipient dependency were associated with higher levels of caregiver burden. Factors 1 and 2 were the most closely related to care-recipient dependency, presenting correlations of -0.6 and -0.5, respectively.

The mean time dedicated to caregiving was 76.46 ±63.81 hours a week, with 35% of participants being 24-hours-a-day caregivers. 

## Discussion

Although most transcultural adaptation studies follow similar methodologies, there is no consensus in the literature regarding how the process should be conducted. This study adopted Beaton's methodological and theoretical model^(^ 15 ^)^, as it offers clear and detailed descriptions on the steps involved in the adaptation process. 

This study was successful in producing an adaptation of the Caregiver Burden Inventory, which showed adequate comprehensibility and was suitable for the assessment of caregivers of older Brazilian persons. However, it is important to note that the levels of education observed in the sample under study were somewhat higher than those observed in other Brazilian studies^(^ 16 ^)^.

Internal consistency values obtained in this study ranged between good and excellent^(^ 1 ^)^, and were similar to those obtained by the original Canadian instrument, the Cronbach's alpha values of which ranged between 0.73 and 0.86^(^ 3 ^)^. In the Chinese validation study, the internal consistency values for factors within the instrument ranged between 0.79 and 0.93^(^ 2 ^)^. The alpha values obtained in this study suggest that the adapted instrument has good internal consistency, assesses domains that are similar to the original instrument and is applicable to the local culture. 

No statistically significant differences were observed between test and retest scores, indicating that the Caregiver Burden Inventory can be re-administered. 

Mean scores in the Caregiver Burden Inventory and Burden Interview could not be directly compared, given that these instruments have different maximum scores. However, correlations were found between all Caregiver Burden Inventory dimensions and scores on the Burden Interview. Therefore, the inventory is comparable to an instrument that is widely used in Brazil - the Burden Interview - in terms of assessing caregiver burden. Items in the instrument produced in this study were somewhat similar to those in the Caregiver Burden Scale^(^ 5 ^)^, but the Caregiver Burden Inventory has more detailed questions regarding care-recipient dependency and the impact of caregiving on the social, family and work lives of caregivers, as seen in dimensions one and four of this instrument. Items in the Family Burden Interview Schedule^(^ 7 ^)^ are likely to be quite distinct from those in this instrument, as it was validated for use with psychiatric patients and its items may be more specific to that population.

When this instrument was validated in China, the mean global scores obtained for the sample (48.8) were higher than those found in this study. This may be because the family caregivers in this study had their own income and could hire outside help, which may not have been possible in the Chinese sample due to the participants' financial statuses. The presence of additional help can reduce burden, as individuals can divide caregiving responsibilities. However, as per the instructions given by the authors of the original instrument, levels of caregiver burden must be assessed based on factor scores, not global scores on the Caregiver Burden Instrument. As in the present study, research in China and Canada found the highest levels of caregiver burden in the time-dependence (15.7 points) and developmental domains (10.2 points). In those two studies, emotional burden was also the factor for which the lowest scores were found^(^ 2 ^-^ 3 ^)^. It is possible that higher levels of time-dependence and personal life burden are associated with the fact that, upon becoming family caregivers, individuals often must drastically change future plans. Perhaps the long hours and the many years dedicated to caregiving by this present sample group influenced the findings in this aspect. Notably, Canadian caregivers also had high levels of time-dependence burden, even though the country has better support structures than either China or Brazil. Future studies into burden-related factors will help clarify these issues.

The satisfactory ICCs found in this study also underscore the stability of burden scores over time. 

Adjustment measures obtained in the confirmatory factor analysis were considered adequate; in other words, the results obtained in the study showed the maintenance of the five components as in the original scale. Correlations between caregiver burden scores and care-recipients' functional capacity showed that higher dependency led to greater caregiver burden scores, especially in factors one and two (time-dependence and personal life burden). Similar results were found in a study where caregivers of older adults with dementia were assessed using the Family Burden Interview Schedule, In that study, the stage of care-recipient dementia was associated with higher levels of caregiver burden, and physical and emotional dedication to caregiving^(^ 17 ^)^. Study conducted in Brazil with family caregivers of seniors who were dependent to some degree in the performance of daily activities, also found that the higher the level of dependence of the elderly individual, the greater the burden the caregiver experiences^(^ 18 ^)^. Levels of emotional burden in this study may have been lower because the sample was not exclusively composed of caregivers of older persons with cognitive impairment. One study conducted in Portugal involved caregivers of family members who had cognitive disabilities and reported greater burden in their relationships with family members compared to those who cared for a family member with no cognitive problems^(^ 19 ^)^. Furthermore, a study that assessed caregivers of individuals with Parkinson's disease using the Caregiver Burden Inventory found associations between higher levels of burden, increased dependency of care-recipients and more symptoms of Parkinson's disease^(^ 20 ^)^. When the present instrument was validated in China, associations were also found between caregiver burden and the functional limitations of care-recipients, with time-dependence and personal life burden being positively associated with the degree of impairment in daily activities^(^ 2 ^)^. 

Many participants in the present study were full time caregivers or provided care for very long hours, which may aggravate stress levels and increase caregiver burden^(^ 17 ^)^. Caregivers also have their own families and often engage in other activities in addition to being full-time caregivers, which leads to increased levels of burden. Most participants in the present study were the children of the care receivers, possibly because, in Latin and Asian societies, the care of older adults traditionally falls upon their family members, and especially their children. A study of this theme conducted in Brazil has shown that being fulltime caregivers for one`s own parents may be one of the greatest difficulties in caregiving^(^ 21 ^)^. The fact that some participants had to spend their own earnings on caregiving expenses may also be related to caregiving burden. However, the present instrument does not assess this particular source of burden, unlike similar instruments that have been validated for use in the Brazilian population and have items or factors that assess the financial burden related to caregiving^(^ 5 ^-^ 7 ^)^. The cultural origins of the present instrument may explain why it does not assess such situations; in developed countries such as Canada, it is unlikely that financial issues would be a source of burden, as caregivers can rely on well-structured support networks for assistance with elder care. This is not the case for Brazilian caregivers and, therefore, one has to take into account a situation that is peculiar to this cultural context where a concern with limited or poor income and the high financial costs of elder care may lead to greater caregiver burden. When, however, the recipient of care does receive an income and significantly contributes to the family budget^(^ 22 ^)^, such additional income may lessen caregiver burden. Therefore, we suggest that future studies conduct more detailed analyses on the financial situation of families and the recipients of care.

## Conclusion

The Caregiver Burden Inventory was adapted and validated for use with caregivers of older persons, and it is validity and reproducibility were demonstrated. The instrument assesses caregiver burden and can provide valuable information regarding the impact of caregiving on numerous domains of the lives of family caregivers. Therefore, it is suggested that nurses assisting families living with dependent older persons make greater use of such instruments in their practice. The use of this instrument in practical contexts such as Primary Health Care units and Home Care agencies can help nurses in developing interventions to prevent caregivers' health problems and improve their quality of life. Future research in the form of longitudinal studies is necessary to further investigate burden-related factors.
